# Red blood cell distribution width and all-cause mortality in low-income populations: a NHANES-based cohort study

**DOI:** 10.3389/fepid.2026.1746886

**Published:** 2026-10-13

**Authors:** Hui Ma, Yu-Han Li, Jin-Da Zhao, Yu-Wei Gu, Ren-Tao Zhu

**Affiliations:** 1 Department of Laboratory Medicine, Fifth People’s Hospital of Kunshan, Suzhou, China; 2 Department of Laboratory Center, The Second Affiliated Hospital, Henan University of Traditional Chinese Medicine, Zhengzhou, China; 3 Department of Intensive Care Medicine, First Affiliated Hospital of Jiamusi University, Jiamusi, China; 4 Suzhou Bozhong Semiconductor Co., Ltd., Suzhou, China; 5 Department of Urology, Fifth People’s Hospital of Kunshan, Suzhou, China

**Keywords:** all-cause mortality, cohort study, low-income groups, NHANES, red blood cell distribution width

## Abstract

**Background:**

Red blood cell distribution width (RDW) may be associated with all-cause mortality. This study aimed to evaluate the relationship between RDW and all-cause mortality in low-income populations. Low-income status was defined as a Family Income to Poverty Ratio (PIR) < 1, consistent with previous studies. All analyses were weighted to account for the complex sampling design.

**Methods:**

This study included 92,062 adults from the National Health and Nutrition Examination Survey (NHANES) 1999–2016 database, with survival follow-up through December 31, 2016. Analyses were restricted to the low-income subgroup and weighted according to the NHANES sampling design to ensure national representativeness. Linear and nonlinear multivariable Cox regression models were used to evaluate the association between RDW and all-cause mortality. Participants with missing data were excluded through complete-case analysis. Statistical analyses were performed using EmpowerStats and R software.

**Results:**

A total of 17,412 participants were included in the final analysis. In the low-income population, RDW showed a nonlinear association with all-cause mortality, characterized by a threshold at 15.9%. Below this threshold, each 1% increase in RDW was associated with a 29% higher risk of mortality (HR = 1.29, 95% CI: 1.20–1.38, *P* < 0.0001), while above 15.9%, the association was no longer statistically significant. Multivariable Cox regression analysis demonstrated that RDW remained significantly associated with mortality after adjustment for potential confounding factors (hazard ratio [HR] = 1.11, 95% confidence interval [CI]: 1.08–1.15, *P* < 0.0001). Specifically, each 1% increase in RDW was associated with an 11% increase in the risk of all-cause mortality.

**Conclusion:**

Elevated RDW was associated with increased all-cause mortality among low-income adults and may indicate a potential prognostic marker for mortality risk in this vulnerable population. However, further studies are needed to validate its clinical predictive utility.

## Introduction

The Family Income to Poverty Ratio (PIR) is an established socioeconomic indicator that reflects household income relative to the federal poverty threshold. PIR is commonly categorized as low income (PIR < 1), middle income (PIR 1–4), and high income (PIR > 4) (1). Numerous studies have demonstrated a significant association between PIR and health status, particularly in relation to chronic diseases and health disparities. For example, lower PIR has been associated with higher rates of childhood morbidity (2), abdominal obesity in women (3), and increased severity of hepatic steatosis in adolescents (4). In addition, children from low-income households have shown a higher prevalence of depression and anxiety (5), as well as poorer lung function compared with those from higher-income households (6). These findings highlight the important role of socioeconomic disadvantage as a determinant of health.

Red blood cell distribution width (RDW), a routine parameter of the complete blood count, reflects variability in erythrocyte size. Although RDW has traditionally been used to support the diagnosis and classification of anemia (7), increasing evidence suggests that elevated RDW is also associated with inflammation, cardiovascular disease, cancer, trauma, and mortality (8, 9). The mechanisms underlying these associations remain incompletely understood. However, RDW may reflect impaired erythropoiesis, oxidative stress, chronic inflammation, and reduced red blood cell survival.

In low-income populations, RDW may be particularly informative because socioeconomic disadvantage is often accompanied by nutritional deficiencies, greater exposure to environmental stressors, chronic inflammatory conditions, and limited access to preventive and medical care. These factors may disrupt erythropoiesis and red blood cell homeostasis, resulting in elevated RDW values. Therefore, RDW may reflect not only hematological abnormalities, such as anemia, but also the broader physiological burden associated with persistent socioeconomic vulnerability.

Previous studies have linked RDW to mortality in patients with hip fractures (10), intracerebral hemorrhage (11), and severe cardiovascular disease (12). Given the established role of RDW in anemia assessment and the higher prevalence of anemia among low-income populations, we hypothesized that RDW would be associated with all-cause mortality in low-income individuals. However, no previous studies have specifically investigated this association. Therefore, the primary aim of this study was to examine the relationship between RDW and all-cause mortality in low-income populations using data from the National Health and Nutrition Examination Survey (NHANES). We further aimed to determine whether RDW was independently associated with mortality risk in this vulnerable population after adjusting for potential confounding factors and underlying health conditions.

## Methods

### Study population

NHANES is a population-based survey designed to assess the health and nutritional status of the civilian, noninstitutionalized population of the United States (13). The survey uses a multistage probability sampling design and collects data through household interviews, physical examinations conducted in mobile examination centers, and laboratory testing. The study population was not restricted to healthy individuals. Instead, it included participants with a wide range of health conditions, thereby reflecting a real-world population. This design enabled us to investigate the association between RDW and mortality among low-income adults, a population in which comorbidities are common. The NHANES protocol was approved by the Ethics Review Board of the National Center for Health Statistics (NCHS), and all participants provided written informed consent.

Low-income status was defined as a PIR < 1, consistent with previous studies (1). Analyses were restricted to this subgroup and incorporated NHANES sampling weights to ensure national representativeness.

A total of 92,062 participants were identified from NHANES 1999–2016. Participants younger than 20 years of age (*n* = 42,550) were excluded. We also excluded participants with missing RDW data (*n* = 4,764), missing mortality data (*n* = 61), missing PIR data (*n* = 3,678), missing body mass index (BMI) data (*n* = 710), or missing covariate data (*n* = 22,887). Missing data were handled using complete-case analysis. Ultimately, 17,412 participants were included in the final analysis. Given the substantial proportion of missing data, complete-case analysis may have introduced selection bias; however, this approach was chosen because missingness was primarily driven by study design and covariate availability rather than outcome-related factors. The sample attrition process is detailed in Figure 1.

**Figure 1:**
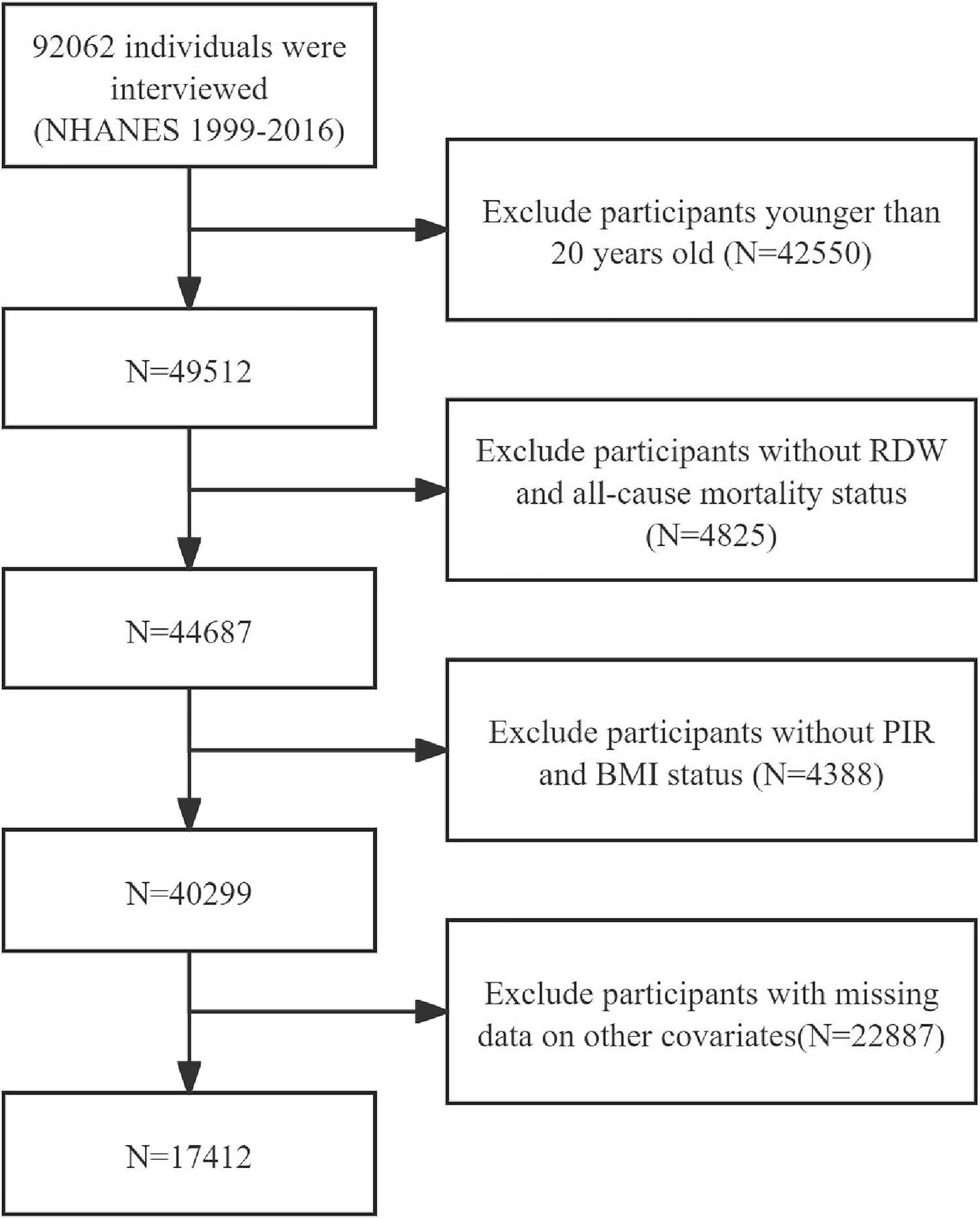
Flow diagram of participant selection.

### Variables

Potential covariates included demographic characteristics, lifestyle factors, medical history, anthropometric measurements, and laboratory indicators. Information on age, sex, race and ethnicity, educational level, smoking status, alcohol consumption, and medical history was obtained through standardized household questionnaires. BMI was measured during the physical examination. RDW was reported as the coefficient of variation (RDW-CV, %). Laboratory indicators, including mean corpuscular volume (MCV), mean corpuscular hemoglobin (MCH), MCH concentration (MCHC), and hemoglobin (HGB), were also collected and included as potential covariates.

### Outcomes

All-cause mortality was determined using records from the National Death Index (NDI) through December 31, 2016, which were linked to the NHANES datasets.

### Statistical analysis

Continuous variables were presented as means ± standard deviation (SD) or medians with interquartile ranges (IQR), as appropriate. Categorical variables are expressed as frequencies and percentages. Differences across RDW quartiles were compared using one-way analysis of variance (ANOVA) for continuous variables and chi-square tests for categorical variables. To investigate the association between RDW and all-cause mortality, generalized additive models (GAMs) were initially applied to explore potential nonlinear relationships (Figure 2). The smoothing degrees of freedom for the GAMs were automatically selected by the software to optimize model fit. Cox proportional hazards regression models were subsequently used to estimate hazard ratios (HRs) and 95% confidence intervals (CIs). The proportional hazards assumption was assessed using Schoenfeld residuals, and no significant violations were observed (all P > 0.05). Three regression models were constructed. Model 1 was unadjusted. Model 2 was adjusted for sociodemographic variables. Model 3 was fully adjusted for demographic (age, sex, race/ethnicity, education), lifestyle (smoking status, alcohol consumption), clinical (BMI, history of cancer, stroke, liver disease, hypertension, diabetes, and kidney disease), and laboratory covariates (MCV, MCH, MCHC, HGB) (Table 1). To evaluate the robustness of the findings, RDW was additionally categorized according to anemia-related criteria, and a P value for trend was calculated. A piecewise Cox regression model was used to evaluate the threshold effect of RDW on mortality (Table 2). The turning point was identified using the threshold effect analysis module of EmpowerStats software, which automatically determines the optimal cutoff value that maximizes model fit within a two-segment Cox regression framework. The statistical significance of the threshold effect was assessed using a log-likelihood ratio test comparing the piecewise model against the standard single-line Cox model. Because the turning point is derived through a data-driven exploratory process, direct confidence intervals for the threshold itself were not estimated. We acknowledge that this method carries a risk of overfitting and emphasize that the identified threshold is exploratory; it requires external validation in independent cohorts before clinical application. All analyses incorporated NHANES sampling weights where appropriate.

**Figure 2:**
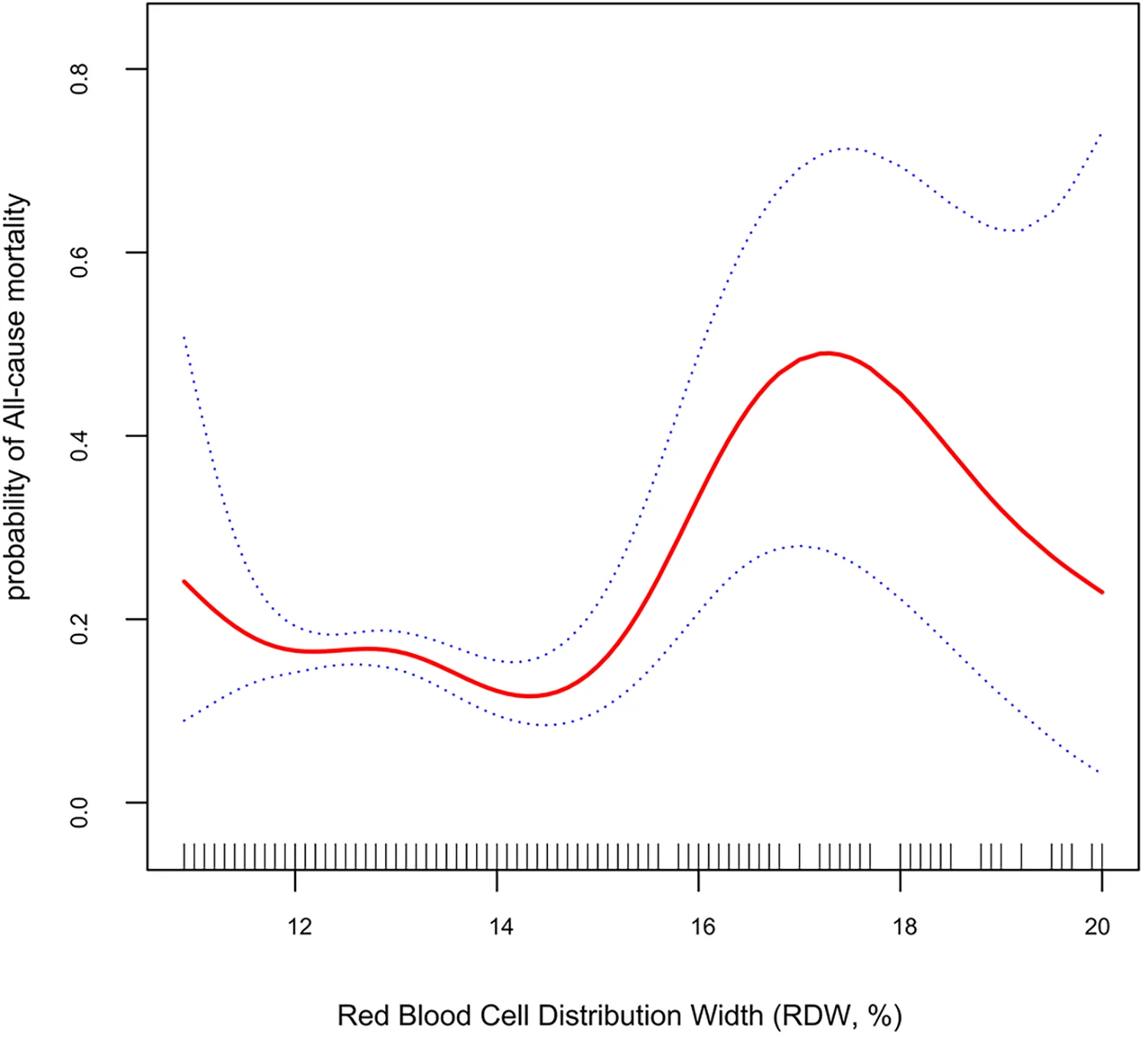
Curve fitting between RDW and mortality. Adjusted for age, sex, education, race, BMI, cancer, stroke, liver disease, smoking status, alcohol consumption, hypertension, diabetes, kidney disease.

**Table 1:** Univariate and multivariate results by cox regression.

Exposure	Non-Adjusted Model	Minimally-Adjusted Model	Fully-Adjusted Model
RDW	1.18 (1.15, 1.21) < 0.0001	1.13 (1.10, 1.16) < 0.0001	1.11 (1.08, 1.15) < 0.0001
RDW quartile			
Q1	1.0	1.0	1.0
Q2	1.37 (1.05, 1.79) 0.0198	1.13 (0.86, 1.47) 0.3893	1.16 (0.88, 1.51) 0.2946
Q3	2.41 (1.88, 3.10) < 0.0001	1.52 (1.18, 1.95) 0.0012	1.50 (1.16, 1.93) 0.0019
Q4	3.56 (2.79, 4.53) < 0.0001	1.95 (1.52, 2.50) < 0.0001	1.88 (1.46, 2.42) < 0.0001
*P* for trend	< 0.0001	< 0.0001	< 0.0001

Data are presented as HR (95% CI) and *P*-value.

**Table 2:** Nonlinearity of RDW versus mortality.

Outcome	HR (95% CI)	*P* value
Model I		
One line effect	1.11 (1.08, 1.15)	< 0.0001
Model II		
Turning point (K)	15.9	
Red blood cell distribution width < K 1	1.29 (1.20, 1.38)	< 0.0001
Red blood cell distribution width > K 2	1.00 (0.94, 1.07)	0.9017
The difference in effect between 2 and 1	0.78 (0.70, 0.87)	< 0.0001
*P* for log-likelihood ratio test		< 0.001

Adjusted for: age, sex, education, race, BMI, cancer, stroke, liver disease, smoking status, alcohol consumption, hypertension, diabetes, kidney disease and laboratory indicators (MCV, MCH, MCHC, HGB).

All statistical analyses were performed using R software (R Project for Statistical Computing; R Foundation for Statistical Computing) and EmpowerStats software (EmpowerStats). HRs and 95% CIs were calculated. A two-sided P value < 0.05 was considered statistically significant.

## Results

### Characteristics of the study population

A total of 17,412 participants were included in this study and were categorized into four quartiles (Q1–Q4) according to their RDW levels. Baseline characteristics across the RDW quartiles are presented in Table 3. Age and BMI increased significantly with increasing RDW quartiles. In contrast, MCV, MCH, MCHC, and HGB levels decreased significantly across the quartiles. In addition, higher RDW quartiles were associated with a greater proportion of females, Black participants, individuals with lower educational attainment, and participants with a history of cancer, stroke, or kidney disease. These findings suggest that RDW is broadly associated with multiple health indicators and disease conditions.

**Table 3:** Baseline characteristics of the study population by RDW quartiles in NHANES 1999-2016.

RDW quartile	Q1	Q2	Q3	Q4	*P*-value
*N*	3,806	4,713	4,491	4,402	
Age (years)	45.33 ± 16.65	49.66 ± 17.20	53.32 ± 17.45	57.13 ± 16.77	< 0.001
BMI (kg/m^2^)	27.56 ± 5.37	28.34 ± 5.99	28.84 ± 6.49	30.23 ± 7.80	< 0.001
MCV (fl)	91.97 ± 4.06	91.38 ± 4.20	90.88 ± 4.83	87.95 ± 7.53	< 0.001
MCH (pg)	31.58 ± 1.58	31.18 ± 1.63	30.85 ± 1.89	29.44 ± 3.04	< 0.001
MCHC (g/dL)	34.34 ± 0.87	34.13 ± 0.83	33.95 ± 0.89	33.42 ± 1.05	< 0.001
HGB (g/dL)	14.80 ± 1.30	14.72 ± 1.34	14.49 ± 1.37	13.67 ± 1.74	< 0.001
Sex					< 0.001
Male	2,255 (59.25%)	2,844 (60.34%)	2,724 (60.65%)	2385 (54.18%)	
Female	1,551 (40.75%)	1,869 (39.66%)	1,767 (39.35%)	2,017 (45.82%)	
Ethnicity					< 0.001
Mexican	678 (17.81%)	827 (17.55%)	655 (14.58%)	469 (10.65%)	
Other Hispanic	206 (5.41%)	306 (6.49%)	341 (7.59%)	308 (7.00%)	
White	2,359 (61.98%)	2,715 (57.61%)	2,375 (52.88%)	1,978 (44.93%)	
Black	357 (9.38%)	602 (12.77%)	865 (19.26%)	1,419 (32.24%)	
Other	206 (5.41%)	263 (5.58%)	255 (5.68%)	228 (5.18%)	
Educational Status					< 0.001
≥ College or above	1,887 (49.58%)	2,095 (44.45%)	1,944 (43.29%)	1,771 (40.23%)	
High school or equivalent	971 (25.51%)	1,228 (26.06%)	1,168 (26.01%)	1,163 (26.42%)	
< High school	947 (24.88%)	1,388 (29.45%)	1,375 (30.62%)	1,465 (33.28%)	
Other	1 (0.03%)	2 (0.04%)	4 (0.09%)	3 (0.07%)	
Cancer					< 0.001
No	3,513 (92.30%)	4,256 (90.30%)	3,947 (87.89%)	3,744 (85.05%)	
Yes	287 (7.54%)	453 (9.61%)	537 (11.96%)	656 (14.90%)	
Unknown	6 (0.16%)	4 (0.08%)	7 (0.16%)	2 (0.05%)	
Stroke					< 0.001
No	3,718 (97.69%)	4,550 (96.54%)	4,254 (94.72%)	4,066 (92.37%)	
Yes	85 (2.23%)	155 (3.29%)	224 (4.99%)	331 (7.52%)	
Unknown	3 (0.08%)	8 (0.17%)	13 (0.29%)	5 (0.11%)	
Liver disease					0.036
No	3,645 (95.77%)	4502 (95.52%)	4,283 (95.37%)	4,148 (94.23%)	
Yes	155 (4.07%)	203 (4.31%)	201 (4.48%)	243 (5.52%)	
Unknown	6 (0.16%)	8 (0.17%)	7 (0.16%)	11 (0.25%)	
Alcohol consumption					< 0.001
No	503 (13.22%)	686 (14.56%)	755 (16.81%)	902 (20.49%)	
Yes	3,301 (86.73%)	4026 (85.42%)	3,735 (83.17%)	3,493 (79.35%)	
Other	2 (0.05%)	1 (0.02%)	1 (0.02%)	7 (0.16%)	
Smoking status					< 0.001
No	386 (10.14%)	406 (8.61%)	328 (7.30%)	314 (7.13%)	
Yes	1,335 (35.08%)	1,767 (37.49%)	1,699 (37.83%)	1,676 (38.07%)	
Other	2,085 (54.78%)	2540 (53.89%)	2,464 (54.87%)	2,412 (54.79%)	
Hypertension					< 0.001
No	2,762 (72.57%)	3,204 (67.98%)	2,710 (60.34%)	2,128 (48.34%)	
Yes	1,038 (27.27%)	1,500 (31.83%)	1,777 (39.57%)	2,268 (51.52%)	
Other	6 (0.16%)	9 (0.19%)	4 (0.09%)	6 (0.14%)	
Diabetes					< 0.001
No	3,445 (90.51%)	4159 (88.25%)	3,825 (85.17%)	3,373 (76.62%)	
Yes	300 (7.88%)	460 (9.76%)	552 (12.29%)	900 (20.45%)	
Unknown	61 (1.60%)	94 (1.99%)	114 (2.54%)	129 (2.93%)	
Kidney disease					< 0.001
No	3,734 (98.11%)	4608 (97.77%)	4,325 (96.30%)	4,124 (93.68%)	
Yes	69 (1.81%)	98 (2.08%)	159 (3.54%)	266 (6.04%)	
Unknown	3 (0.08%)	7 (0.15%)	7 (0.16%)	12 (0.27%)	

Data are presented as mean ± SD/n (%). For continuous variables, the Kruskal–Wallis rank-sum test was used; Fisher's exact test was used for categorical variables with expected cell counts < 10.

**Table 4:** Effects of factors on mortality measured by univariate analysis.

Variables	Statistics	HR (95% CI)	*P* value
Age (years) tertile			
< 45	6,461 (37.11%)	1.0	
45–64	6,255 (35.92%)	4.90 (4.27, 5.63)	< 0.0001
≥ 65	4,696 (26.97%)	21.64 (18.98, 24.67)	< 0.0001
BMI category			
Underweight (< 18.5)	307 (1.78%)	1.0	
Normal (18.5–24.9)	4,801 (27.80%)	0.62 (0.51, 0.77)	< 0.0001
Overweight (25.0–29.9)	6,023 (34.87%)	0.61 (0.50, 0.75)	< 0.0001
Obese (≥ 30.0)	6,140 (35.55%)	0.59 (0.48, 0.72)	< 0.0001
Sex			
Male	10,208 (58.63%)	1.0	
Female	7,204 (41.37%)	0.70 (0.65, 0.75)	< 0.0001
Race and ethnicity			
Mexican	2,629 (15.10%)	1.0	
Other Hispanic	1,161 (6.67%)	0.94 (0.78, 1.14)	0.5578
White	9,427 (54.14%)	1.73 (1.56, 1.91)	< 0.0001
Black	3,243 (18.63%)	1.58 (1.41, 1.79)	< 0.0001
Other	952 (5.47%)	0.83 (0.66, 1.04)	0.0991
Educational status			
< High school	7,697 (44.21%)	1.0	
High school or equivalent	4,530 (26.02%)	1.22 (1.12, 1.33)	< 0.0001
≥ College or above	5,175 (29.72%)	1.67 (1.55, 1.79)	< 0.0001
Other	10 (0.06%)	6.11 (3.17, 11.77)	< 0.0001
Laboratory indicators			
MCV(fl)	90.51 ± 5.58	1.05 (1.04, 1.06)	< 0.0001
MCH(pg)	30.74 ± 2.27	1.06 (1.04, 1.07)	< 0.0001
MCHC(g/dL)	33.95 ± 0.97	0.80 (0.77, 0.83)	< 0.0001
HGB(g/dL)	14.41 ± 1.52	0.87 (0.86, 0.89)	< 0.0001
Cancer			
No	15,460 (88.79%)	1.0	
Yes	1,933 (11.10%)	2.94 (2.72, 3.17)	< 0.0001
Other	19 (0.11%)	1.08 (0.40, 2.88)	0.8799
Stroke			
No	16,588 (95.27%)	1.0	
Yes	795 (4.57%)	3.81 (3.43, 4.22)	< 0.0001
Other	29 (0.17%)	3.24 (1.88, 5.59)	< 0.0001
Liver disease			
No	16,578 (95.21%)	1.0	
Yes	802 (4.61%)	1.52 (1.32, 1.74)	< 0.0001
Other	32 (0.18%)	1.89 (1.04, 3.41)	0.0356
Alcohol consumption			
No	2,846 (16.35%)	1.0	
Yes	14,555 (83.59%)	0.68 (0.63, 0.73)	< 0.0001
Other	11 (0.06%)	3.31 (1.38, 7.99)	0.0076
Smoking status			
No	1,434 (8.24%)	1.0	
Yes	6,477 (37.20%)	1.57 (1.33, 1.85)	< 0.0001
Other	9,501 (54.57%)	2.50 (2.13, 2.93)	< 0.0001
Hypertension			
No	10,804 (62.05%)	1.0	
Yes	6,583 (37.81%)	2.78 (2.61, 2.97)	< 0.0001
Other	25 (0.14%)	1.95 (0.87, 4.34)	0.1036
Diabetes			
No	14,802 (85.01%)	1.0	
Yes	2,212 (12.70%)	2.63 (2.44, 2.85)	< 0.0001
Other	398 (2.29%)	1.88 (1.55, 2.28)	< 0.0001
Kidney disease			
No	16,791 (96.43%)	1.0	
Yes	592 (3.40%)	3.00 (2.65, 3.41)	< 0.0001
Other	29 (0.17%)	2.44 (1.42, 4.21)	0.0013

HR, hazard ratio; CI, confidence interval. Results were derived from univariate Cox regression analyses, with each variable entered individually into the model without adjustment for other covariates.

### Univariate analysis of the association between variables and mortality

We performed univariate analyses to identify potential confounding factors and to examine the associations between individual variables and mortality. The results showed that older age, lower educational attainment, non-White race, and the presence of cancer, stroke, liver disease, hypertension, diabetes, or kidney disease were associated with a higher risk of mortality (Table 4).

### Multivariate analysis of RDW and mortality

We evaluated the association between RDW and all-cause mortality in low-income populations using three progressively adjusted models (Table 1). In additional analyses, RDW was categorized into quartiles based on its distribution in the NHANES database, and the association between RDW categories and all-cause mortality was further examined. RDW was also analyzed as a continuous variable. The fully adjusted model showed that, after controlling for potential confounding factors, each 1% increase in RDW was associated with an 11% increase in the risk of mortality (HR = 1.11, 95% CI: 1.08–1.15, *P* < 0.0001). Compared with participants in the lowest RDW quartile, those in higher quartiles had a significantly increased risk of all-cause mortality (e.g., HR = 1.88, 95% CI: 1.46–2.42, *P* < 0.0001).

Model 1: Crude model, no covariates were adjusted.
Model 2: Minimally adjusted model, adjusted for sociodemographic variables including age, sex, education, and race.
Model 3: Fully adjusted model, adjusted for all covariates in Model 2 plus clinical and lifestyle factors including BMI, smoking status, alcohol consumption, history of cancer, stroke, liver disease, hypertension, diabetes, kidney disease, and laboratory indicators (MCV, MCH, MCHC, HGB).

### Identification of nonlinear relationship

Figure 2 depicts the dose-response relationship between RDW and all-cause mortality estimated by generalized additive models. The curve indicates a nonlinear association, with mortality risk increasing steeply at lower RDW values and plateauing at higher levels. Piecewise Cox regression analysis identified a threshold at RDW = 15.9% (Table 2). Below this threshold, each 1% increase in RDW was associated with a 29% higher mortality risk (HR = 1.29, 95% CI: 1.20–1.38, *P* < 0.0001), whereas no significant association was observed above 15.9% (HR = 1.00, 95% CI: 0.94–1.07, *P* = 0.9017).

## Discussion

Our study identified a significant nonlinear association between RDW and all-cause mortality in low-income populations. Specifically, each 1% increase in RDW was associated with an 11% increase in the risk of mortality (HR = 1.11; 95% CI: 1.08–1.15; *P* < 0.0001). These findings raised the hypothesis that RDW might be associated with prognostic relevance in this demographic; however, given the observational study design, baseline RDW should not be interpreted as an established clinical prediction tool, and any potential clinical application would require prospective validation. The inclusion of a diverse, real-world low-income population, rather than an exclusively healthy cohort, enhances the generalizability and clinical relevance of the findings. This indicates that the association between RDW and mortality persists even in populations with a higher burden of pre-existing conditions, which is characteristic of low-income groups. To the best of our knowledge, this is the first study to report this association specifically in low-income populations.

While statistically significant, the magnitude of this association (HR 1.11 per 1% increase in RDW) is modest. In the context of an observational study, this effect size may partly reflect residual confounding from unmeasured or imprecisely measured variables (e.g., unmeasured inflammatory markers, iron or nutritional deficiencies, or subclinical conditions not captured by self-reported medical history). Therefore, the observed HR should not be interpreted as an independent causal effect of RDW on mortality. Nevertheless, because RDW is routinely available at no additional cost in standard complete blood count testing, even a modest association may have public health relevance at the population level in low-income settings where access to advanced diagnostics is limited. The value of RDW in this setting may lie not in high-precision individual risk prediction, but as an accessible signal that prompts closer clinical monitoring or further evaluation.

RDW, a measure of erythrocyte size variability, is not only a diagnostic marker for anemia but also a potential prognostic indicator for a range of diseases, including cardiovascular diseases (14), cancers (15), and inflammatory conditions (16, 17). In low-income populations, the biological mechanisms underlying elevated RDW are likely multifactorial. Individuals in these groups frequently experience chronic nutritional deficiencies (e.g., iron, folate, and vitamin B12), greater exposure to environmental stressors, a higher prevalence of chronic inflammatory conditions, and limited access to healthcare and preventive services. These factors can collectively impair erythropoiesis and disrupt red blood cell homeostasis, leading to increased red blood cell destruction or ineffective production, which is reflected as elevated RDW. Thus, in low-income settings, RDW may function as an integrative biomarker reflecting not only hematological abnormalities but also the broader physiological consequences of sustained socioeconomic disadvantage. This perspective suggests that RDW may represent a valuable yet underexplored indicator of overall health vulnerability and mortality risk in this population.

The socioeconomic vulnerability of low-income populations is central to understanding the association between RDW and all-cause mortality. Poverty directly impairs nutritional status and access to healthcare, and it indirectly increases health risks by elevating the prevalence of chronic conditions such as kidney stones (18), HPV infection (19), and musculoskeletal disorders (20). Prior studies have consistently reported higher all-cause mortality and poorer musculoskeletal outcomes in lower-income groups. Our findings are consistent with previous evidence linking elevated RDW to adverse outcomes in patients with sepsis (21), traumatic brain injury (22), hip fracture (23), and acute aortic dissection (24). Unlike most prior studies focusing on specific diseases or hospitalized populations, we examined a socioeconomically disadvantaged subgroup within the NHANES. Given that low-income adults typically carry a higher burden of comorbidities and environmental stressors, elevated RDW may be associated with underlying physiological stress in this population, although this interpretation remains speculative and does not imply a causal relationship. Our results further highlight the substantial influence of socioeconomic factors on health outcomes.

We adjusted for an extensive set of demographic, lifestyle, clinical, and laboratory covariates; however, residual confounding remains a possibility. For example, iron deficiency, chronic infections, and subclinical inflammatory states—which are prevalent in low-income populations and may elevate RDW—were not fully captured in NHANES. If such factors are strongly associated with both elevated RDW and mortality, they may have inflated the observed HR of 1.11. Conversely, measurement error in covariates (e.g., self-reported smoking or alcohol use) could also bias the effect estimate in either direction. Consequently, the clinical significance of RDW should be viewed cautiously: it is unlikely to be a high-precision individual risk predictor, but it may serve as a useful population-level screening indicator in resource-limited settings.

We observed a nonlinear association between RDW and mortality, characterized by a threshold effect at 15.9%. Below this threshold, each 1% increase in RDW was associated with a significantly higher mortality risk (HR = 1.29, 95% CI: 1.20–1.38, *P* < 0.0001). However, above 15.9%, the association was not statistically significant (HR = 1.00, 95% CI: 0.94–1.07, *P* = 0.9017). This threshold effect may reflect a saturation phenomenon, in which the deleterious biological processes associated with elevated RDW, such as chronic inflammation, ineffective erythropoiesis, and oxidative stress, reach a plateau (25). Beyond this level, additional variability in erythrocyte size may no longer provide incremental prognostic information.

Alternatively, the attenuation of the association at higher RDW levels may be explained by competing risks. Individuals with markedly elevated RDW often have severe underlying conditions, such as advanced malignancy, end-stage renal disease, or high-grade myelodysplastic syndromes, in which mortality is primarily driven by the underlying disease rather than RDW itself (26). We acknowledge that a formal competing risks analysis was not performed in this study. Given the high mortality rate and the age distribution of our low-income population, different causes of death likely compete, and this could influence the observed association between RDW and all-cause mortality, particularly at higher RDW values. While our current Cox regression models estimate the hazard of all-cause mortality, they do not specifically account for the probabilities of different types of events (e.g., cardiovascular death vs. cancer death) in the presence of other events. This represents a limitation of our current analysis. Future studies could benefit from employing specific competing risks models (e.g., Fine-Gray models) to disentangle the effects of RDW on cause-specific mortality in this vulnerable population. In addition, the relatively small number of participants with RDW values above 15.9% may have limited statistical power in this range, potentially contributing to the observed flattening of the association (27). Importantly, the identified threshold of 15.9% should be interpreted as exploratory and data-driven. It has not been externally validated and should not be considered a clinically established cutoff. Further studies in independent cohorts with larger numbers of individuals with markedly elevated RDW are required to confirm this finding.

To ensure robust estimates, we adjusted for multiple potential confounders, including age, sex, education, race and ethnicity, BMI, smoking status, alcohol consumption, and histories of cancer, stroke, hypertension, diabetes, kidney disease, and liver disease. Several limitations should be acknowledged. First, the complete-case analysis resulted in the exclusion of 74,650 individuals (81.1% of the initial sample) due to missing data on key covariates. Although we applied NHANES sampling weights to enhance national representativeness, this high exclusion rate may compromise the generalizability of our findings and may introduce selection bias if the missingness is not completely at random. Nonetheless, the association between RDW and all-cause mortality remained consistently significant across all three progressively adjusted models, with stable hazard ratios and narrow confidence intervals. This consistency suggests that the observed effect is unlikely to be entirely attributable to the excluded participants. In addition, the biological plausibility of the RDW-mortality relationship has been extensively documented in previous studies across diverse populations, which further supports the credibility of our results. Nevertheless, future studies with more complete data collection strategies are warranted to externally validate our findings in low-income populations.

## Conclusion

In this nationally representative cohort of low-income adults, we identified a significant association between elevated RDW and increased all-cause mortality, with evidence of a nonlinear relationship characterized by a threshold at 15.9%. Below this threshold, each 1% increase in RDW was associated with a 29% higher risk of mortality, whereas no significant association was observed above 15.9%. These findings suggest that RDW is associated with mortality risk in low-income adults, although the modest effect size (HR 1.11 per 1% increase) and the potential for residual confounding limit its interpretation as a high-precision individual prediction tool. RDW may hold value as an accessible, low-cost population-level indicator for risk stratification in resource-limited settings, but its clinical predictive utility requires prospective validation before implementation.

## Data Availability

The original contributions presented in the study are included in the article/Supplementary Material, further inquiries can be directed to the corresponding authors.
